# Novel Femto Laser Patterning of High Translucent Zirconia as an Alternative to Conventional Particle Abrasion

**DOI:** 10.3390/dj9020020

**Published:** 2021-02-08

**Authors:** Emmanouil-George C. Tzanakakis, Anastasia Beketova, Lambrini Papadopoulou, Eleana Kontonasaki, Ioannis G. Tzoutzas

**Affiliations:** 1 School of Dentistry, National and Kapodistrian University of Athens, 2 Thivon Str, Goudi, 11527 Athens, Greece; tzanakak@dent.uoa.gr (E.-G.C.T.); tzoudent@dent.uoa.gr (I.G.T.); 2 Department of Prosthodontics, School of Dentistry, Faculty of Health Sciences, Aristotle University of Thessaloniki, 54124 Thessaloniki, Greece; anastasiabeketova@yahoo.com; 3 School of Geology, Faculty of Sciences, Aristotle University of Thessaloniki, 54124 Thessaloniki, Greece; lambrini@geo.auth.gr; 4 Center for Interdisciplinary Research and Innovation (CIRI-AUTH), Balkan Center, 57001 Thessaloniki, Greece

**Keywords:** zirconia ceramic, resin cement, shear bond strength, laser, surface treatment

## Abstract

Background: currently applied surface treatments for zirconia bonding may create undesired microcracks and surface flaws. The aim of the present study was to evaluate the efficacy of alternative surface treatments on the shear bond strength of high translucency zirconia to 10-Methacryloyloxydecyl dihydrogen phosphate (MDP)-containing resin-based cement. Methods: fifty disk-shaped specimens (10 mm × 5 mm) were fabricated from a commercial yttria-stabilized zirconia with 5 mole% yttrium oxide tetragonal zirconia polycrystal (5Y-TZP), and underwent air-abrasion with alumina particles (50 μm-AL50 and 90 μm-AL90), glass beads (GB 10–60 μm), and ablation with femtosecond laser (FEMTO). Shear bond strength was evaluated with a universal testing machine under a crosshead speed of 0.5 mm/min until fracture. Fracture type was evaluated with an optical stereomicroscope. Differences among groups were evaluated by one-way ANOVA and Bonferroni pairwise comparison tests (*p* < 0.05). Results: the highest shear bond strength values were presented by the laser treated group (23.97 ± 3.7 MPa). No statistically significant differences were found among the Cl, Al50, Al90 and FEMTO groups. The lowest mean value was presented by the glass-beads treated group (11.93 ± 2.88 MPa) which was significantly lower compared to all other groups (*p* < 0.001). Conclusions: under the limitations of this in vitro study, femtosecond laser treatment of High-translucent monolithic zirconia (HTZ) ceramics is a promising alternative method for the mechanical retention of resin cements.

## 1. Introduction

In recent years, dental manufacturers have introduced a new generation of zirconia ceramics that are suitable for tooth reconstruction in the esthetic zone. High-translucent monolithic zirconia (HTZ) has gained much interest from patients with esthetic demands because of its superior optical properties. In clinical practice, high-translucent zirconia has been used to produce monolithic anterior crowns and ultrathin restorations. Its higher translucency is achieved by a slight increase in the Y_2_O_3_ content (higher than 3 mol-%), which also increases the percentage of the transparent cubic-phase [1,2]. On the other hand, HTZ materials possess significantly lower flexural strength (550–800 MPa instead of 900–1400 MPa), and even poorer adhesive behavior with resin based cements as compared to conventional zirconia [3,4,5]. Decementation of crowns, especially in the anterior area, can compromise the whole clinical outcome and cause patient dissatisfaction, whereas in case of ultrathin HTZ restorations, cementation failure can easily result in chipping or fracture, as resin bonding only provides them with limited strength.

There are limited scientific data about the bonding strength of translucent zirconia subjected to different surface treatments. As other types of zirconia, HTZ possesses a dense polycrystalline structure with no vitreous phase, which makes its surface processing quite complicated. Methods such as airborne abrasion with alumina particles [6], tribochemical silica coating [7], deposition of low fusing porcelain [8], plasma coating with hexamethyldisiloxane [9], laser surface ablation [10,11], selective infiltration etching [12], and zirconia primers/adhesives [13] and many others have been proposed for conventional zirconia.

Currently, the most reliable bonding protocols for zirconia is the combination of air-abrasion with alumina (Al_2_O_3_) or silica-coated alumina (Al_2_O_3_/SiO_2_) particles and further surface conditioning with an MDP-containing primer [14,15]. Abrasive alumina particles of different sizes are used to remove impurities and increase its surface roughness, thereby providing mechanical interlocking with cement. In turn, MDP-containing primer can chemically bond to the zirconia surface through zirconium phosphate formation. Recently, Salem et al. [16] reported promising results for translucent zirconia adhesive strength when using alumina air-particle abrasion combined with an MDP-containing adhesive. Another technique, air abrasion with silica-coated alumina particles, known as tribochemical silica coating, leaves the zirconia surface with a thin silica layer that is able to react with silane. In this respect, Ruales-Carrera et al. [5] found that tribochemical treatment with Al_2_O_3_/SiO_2_ particles was effective at increasing the bond strength of both conventional and highly translucent zirconia. On the other hand, recent in vitro studies reported that airborne-particle abrasion with coarse particles may create microcracks, initiating fractures on the zirconia surface [17,18]. For this reason, there is a strong need for investigation of alternative surface conditioning methods for zirconia, particularly, for the more brittle HTZ materials.

Recently, it has been reported that air blasting of the zirconia surface with glass beads can improve the bond strength between zirconia and resin cement [19]. The fusion of glass particles on a zirconia surface has a two-fold effect; it creates a chemical bond between zirconia and cement through the silane bonding and modifies surface topography increasing bond strength [9,20]. However, airborne abrasion with glass beads has not been applied to HTZ materials so far.

Another promising method for modifying the zirconia surface, is application of a laser. Laser surface treatment is rapid, non-contact and precise, as it allows one to obtain high-resolution features, down to the nanoscale. Several short pulse lasers such as Nd:YAG, Er:YAG, and Er,Cr:YSGG continuous wave carbon dioxide (CO_2_), have been suggested for zirconia surface treatment [21,22,23]. However, Er:YAG and CO_2_ lasers can cause surface microcracking [22,24] which might reduce the flexural strength. On the other hand, femtosecond lasers can produce ultrashort pulses of high intensity and ablate material only superficially, without causing thermal damage. To achieve a desired surface pattern, different types of surface craters can be created through appropriate software. Femtosecond laser application creates rough zirconia surfaces without triggering phase transformations [25,26]. Moreover, in our previous study it was demonstrated that femtosecond laser patterning of HTZ surface resulted in a rough surface pattern in laser affected areas and the laser-induced grooves presented a repetitive morphology with parallel lines [27].

Based on the aforementioned, the aim of the present study was to evaluate the efficacy of novel surface treatments—air-particle abrasion with glass beads and femtosecond laser ablation—on the shear bond strength of HTZ to resin-based cement. The research hypothesis was that different surface treatments provide the same shear bonding strength and fracture mode between zirconia and resin-based cement.

## 2. Materials and Methods

For the experiments, white colored blocks of commercial yttria-stabilized zirconia 5Y-TZP (92% ZrO_2_, 5.2% Y_2_O_3_, HfO_2_ < 4%, and Al_2_O_3_ < 0.5%, SiO_2_ < 1%) (Bruxzir HT 2.0, Glidewell, Newportbeach, CA, USA) (LOT HT–BZ0014192) were used. For air abrasion, Al_2_O_3_ particles of size 50 μm (Luoyang, Yannuo, China), Al_2_O_3_ particles with size 90 μm (Danville San Ramon, CA, USA) and glass beads (Luoyang, Yannuo, China) with particle diameter of 10–60 μm were used. Commercially available methylacrylate-based dual-cured resin cement Panavia V5 together with Clearfil ceramic primer plus (Kuraray, Okayama, Japan) were chosen as experimental materials because they incorporate MDP functional monomers.

Fifty disk-shaped specimens with diameter 10 mm and height 5 mm were fabricated using Computer Aided Design/Computer Aided Manufacturing (CAD/CAM). Then, the specimens were impregnated in epoxy resin (Epofix, Struers, Denmark) using Plexiglas molds of 25 mm height, 12 mm internal diameter and 16 mm external diameter and were kept for 12 h under atmospheric pressure until the complete polymerization of epoxy resins. After removal of the specimens from the molds they were ground in a rotary motion metallurgical grinding apparatus at speed of 200 rpm and water irrigation. Grinding and polishing of specimens was performed under constant pressure using metallic holder with 220 and 1200 grit silicon carbide grinding discs for 10 min. In order to check for surface defects during construction and processing, each specimen was examined using a stereo microscope (M80, Leica, Weltzar, Germany) connected to a PC with the appropriate software at magnifications of 7.5× to 60×. Specimens with surface defects were removed and replaced with new ones. Finally, all specimens were ultrasonically cleaned in a pure ethyl alcohol for 15 min, dried with a mild air stream and divided into 5 equal groups (N = 10) based on surface treatment, as following:

(a). CL group: Control group with no further processing after polishing;
(b). AL50 group: air-abraded by 50 μm alumina particles;
(c). AL90 group: air-abraded by 90 μm alumina particles;
(d). GB group: air-abraded by glass beads of various diameters of 10–60 μm;
(e). FEMTO group: in which parallel grooves of 50 μm in the central area of 5 × 5 mm were processed by femtosecond laser ablation.

The air abrasion process for the AL50, AL90 and GB groups was performed using an intraoral sandblaster device (Microetcher IIA, Danville, CA, USA), equipped with a 0.048 diameter nozzle, at the air pressure 36 psi (2.5 bar) for 10 s and distance of 10 mm and incidence angle of 45° to the free zirconia surface. A special holder was fabricated to maintain exact distance and incidence angle in all air abraded specimens.

For the experiments, a Yb: KGW laser was used, emitting linearly polarized light with central wavelength of 1026 nm, pulse duration 170 fs and repetition rate of 1 KHz. The laser beam was focused on the sample through 100 mm achromatic convex lens while the estimated Gaussian spot size, had 35 μm diameter, as measured with a CMOS camera. The zirconia samples were mounted in a 3-axis support and movement device and placed vertically to the incident beam. Laser radiation was emitted under normal environmental conditions with normal incidence direction. The designed surface pattern included an area of 5 mm × 5 mm and consisted of continuous horizontal line scans with 50 μm line spacing. For the surface processing the zirconia samples the laser parameters were: laser fluence, F = 9.6 J/cm^2^ and sample scanning speed, v = 1 mm/s.

At first, each specimen was coated by one component adhesive primer (Clearfil Ceramic Primer plus) using an applicator brush, as described by the manufacturer. For repeatability and precision of the bonding procedures, five special two-piece teflon guides with a center hole (3 × 3 mm) were constructed for zirconia specimens. Then, the resin-based cement (Panavia V5) was injected using self-mixing syringe in the center of each teflon guide until the empty space was filled. After 1 min the cement was photo-activated using light emitting diode-LED (T-LED, Anthos, Imola, Italy) with output power 800 mW/cm^2^ for 20 s. After polymerization and complete removal of the teflon guides, the specimens were kept in deionized water at 37 °C in a temperature controlling apparatus for one week.

For shear bond strength test a universal testing machine (Monsanto Tensometer 10, Swindon, UK) was used operating in shear mode with a crosshead speed of 0.5 mm/min until fracture of the zirconia/cement interface. Maximum values of shear strength (peak points), expressed as N, were recorded for each specimen. In order to calculate the maximum shear stress, these values were conversed to MPa, using the equation P = F/S, where F is the force in Newton (N) and S = πR^2^ the bonded surface.

For the evaluation of the type of fracture, the detached surfaces were photographed using an optical stereomicroscope at magnifications of 10–60X. Quantification of the remaining ceramic mass or resin-based cement on the surface of zirconia was done with the help of a PC software program (Adobe Systems, San Jose, CA, USA). Fracture was defined as adhesive in cases where more than 75% of the zirconia surface was visible. The fracture was defined as cohesive when more than the 75% of the core surface was covered with resin. All other cases were defined as mixed fractures. For further evaluation of the debonded surfaces and elemental analysis, Scanning Electron Microscopy (JEOL J.S.M. 840A, Tokyo, Japan) and Energy Dispersive Spectroscopy (Oxford INKA-300) were applied. A vacuum evaporator (JEOL-4X) was used to coat the surfaces with a carbon coat of 200 Å for SEM-EDS evaluation. Shear bond strength values were analyzed for normality and homogeneity of variance with Kolmogorov–Smirnof and Levene tests, respectively, while differences among groups were evaluated by one-way ANOVA and Bonferroni multiple comparison test. The results of failure type were also were also evaluated with one-way ANOVA. Statistical analysis was performed with the IBM Statistics SPSS 20.0 software with significance level set at *p* < 0.05.

## 3. Results

**Figure 1:**
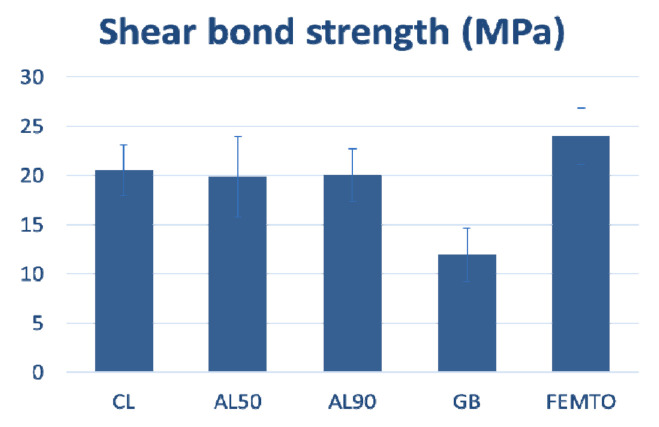
Mean values and standard deviation (bars) of shear bond strength values.

The results of the maximum shear strength and the standard deviations in MPa of the five experimental groups are presented in Figure 1. The ranking of groups in decreasing shear bond strength were: FEMTO > AL90, AL50, CL > GB.

According to the ANOVA analysis, no statistically significant differences were found among the Cl, AL50, AL90 and FEMTO groups (F: 16.638, df: 4, *p* < 0.001). The lowest mean value was recorded for the GB group (11.93 ± 2.88 MPa) which was statistically significantly different compared with all other groups (*p* < 0.001) (Table 1).

The highest mean shear bond strength values were presented by the FEMTO group (23.97 ± 3.7 MPa) which was statistically significantly different only compared to the GB group (*p* < 0.001). According to adhesive failure modes (ADFM) analysis FEMTO, Cl, AL50 and AL90 groups presented significantly lower ADFM values than the GB group (Table 2).

**Figure 2:**
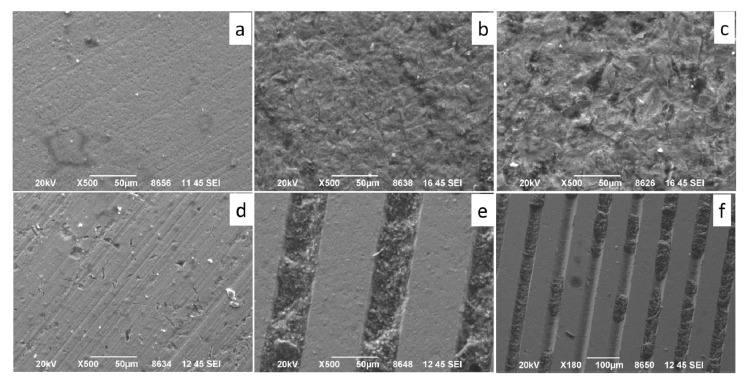
SEM microphotographs of fractured surfaces. (**a**) control (CL group), (**b**) air-borne microparticles of 50 μm alumina (AL50 group). (**c**) air-borne microparticles of 90 μm alumina (AL90 group), (**d**) air-borne microparticles of glass beads (GB group) and (**e**) laser pattern of parallel grooves of 50 μm (FEMTO group), (**f**) lower magnification microphotograph of FEMTO group showing cement remnants in all microgrooves.

SEM-EDS analysis showed the surface pattern composed of parallel grooves filled with resin-based cement of the FEMTO group specimens (Figure 2).

**Figure 3:**
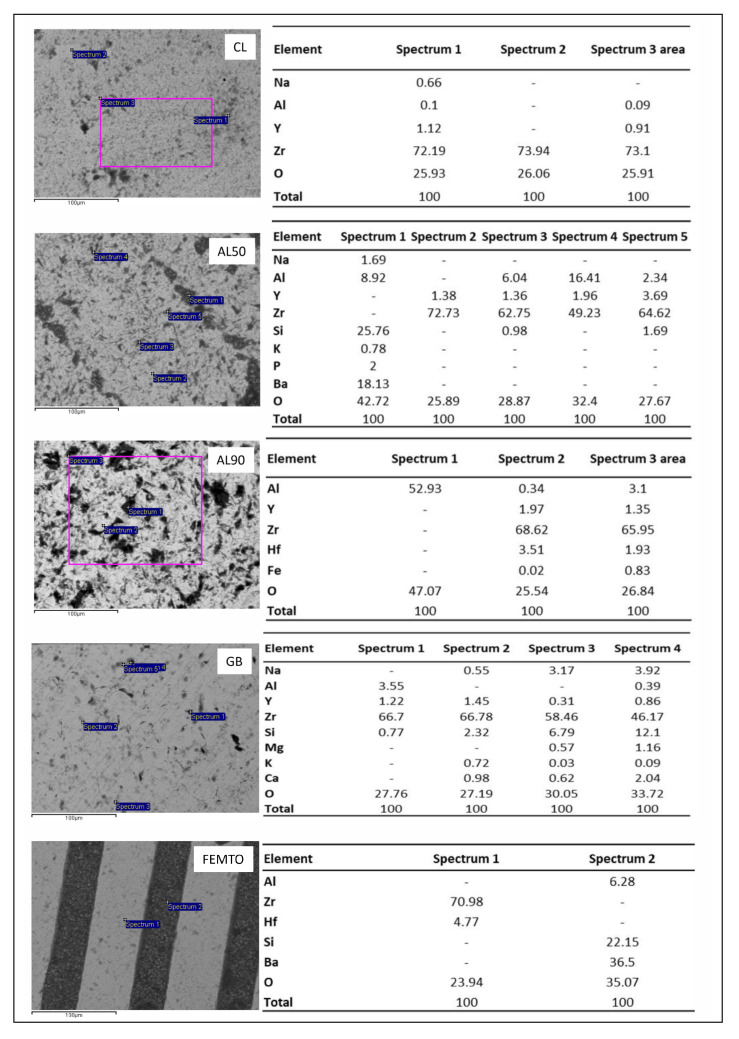
Left: SEM Backscattered microphotographs. Right: EDS compositional analysis from the respec-tive microphotographs.

The SEM microphotographs of AL90 and AL50 samples show irregular rough surface with residues of cement on it (Figure 3). The surface of AL90 appeared to be rougher as compared to the specimens of AL50 group, GB and CL. EDS analysis showed the presence of Al_2_O_3_-rich phase entrapped within the surface irregularities in AL50 and AL90 specimens. Finally, the surface roughness GB treated specimens remained unchanged and similar to the control group, while the presence of small superficial Si-containing particles was detected by EDS (Figure 3).

## 4. Discussion

In the presented study five different surface treatments were applied to novel high-translucent zirconia and shear bonding strength with resin-based cement was evaluated. The results showed that the GB group presented statistically significant differences compared with the FEMTO, CL, AL50, AL90 groups in shear bond strength and fracture type, and that there were no statistically significant differences among specimens of FEMTO, CL, AL50 and AL90 groups, thereby partially rejecting the research hypothesis.

The highest average values of shear bond strength (23.97 ± 3.7 MPa) were observed in specimens of the FEMTO group, which presented a moderated surface roughness of 307.3 ± 32.7 nm [27], which was similar to AL50 (AL50 = 340.3 ± 49.2 nm) but significantly higher compared to CL (CL = 73.9 ± 10.7 nm) [27]. Similar results were obtained by Vicente Prieto et al. [28], who reported superior values of Shear Bond Strength (SBS) in specimens that underwent femto-laser surface patterning as compared with airborne-particle abrasion (APA) and tribochemical coating of conventional zirconia. At the same time, the FEMTO group also presented mostly mixed type fracture, and similar percentage of adhesive failure modes (ADFM) compared with CL, AL50 and AL90 groups. In fact, the SEM analysis presented penetration of the cement inside the grooves and adhesive decementation at all other areas. Moreover, the presence of the element barium (Ba) in high percentage (36.5%) proves the high percentage of resin cement in the laser groove area. The results of the fracture type suggest that the laser treated surface resulted in mechanical interlocking that was responsible for the high acquired SBS values. However, as seen in lower magnification (Figure 3e), some areas in the grooves were not covered by resin cement. One possible explanation is that the rheological properties of the resin cement hampered the complete penetration of the material in the grooves. In addition, inhomogeneous diffusion of the primer inside the retentive laser lines may have contributed to this result. This hypothesis is also supported by the unexpectedly high shear strength values of the control group which are mainly based on chemical activation rather than micromechanical retention. The depth, width and roughness of the retentive grooves need further evaluation to define the optimum conditions. Additionally, shallower or wider grooves could perhaps allow better flow of the primer. Another explanation is that cohesive strength of the resin cement surpassed the shear bond adhesive potential and resin cements remnants could have been detached during shear stressing.

Processing of HTZ in femtosecond regime enabled precise micropatterning and creation of parallel retention microgrooves, to obtain extra space for cement. SEM images revealed increased microroughness within the grooves due to ablation while the untreated surface remained smooth and free of microcracks, pores and signs of melting. These findings suggest that the ultrashort pulses with high intensity applied in the present study caused ablation only within laser generated plasma, without causing thermal damage to the adjacent regions due to heat conduction; a phenomenon usually observed with conventional lasers (nanosecond or longer pulses) [25].

According to recent studies, both irradiation patterns [29] and different angulations [26,30] of the laser affect the bond strength between the ceramic surface and resin-based material. Vincente et al. [31] produced two uniform surface patterns with parallel grooves with depth of 20 μm using scanning steps of 20 and 40 μm and found no significant differences in shear bond strength between groups. In accordance with our results, the authors observed mostly mixed types of failures. Akpinar et al. [26] used different beam angles (45°, 60°, 75° and 90°) on zirconia surfaces and reported smaller bond strength values with the 90° beam angle. Yucel et al. [30] also reported better results with the 30° laser beam angle, which were attributed to more retentive areas with this beam inclination that resulted in an increase in the total contact area. So far, further improvements in the bond strength of laser processed HTZ ceramics could be achieved by optimization of laser surface patterning.

Air-borne particle abrasion was applied to HTZ using alumina particles of different sizes (50 μm and 90 μm) and GB. The AL50 and AL90 groups had similar bond strength values and similar types of fracture, covering a high percentage of the zirconia surface with resin material, suggesting that surface roughness profile did not significantly affect the shear strength. The obtained values of bond strength for alumina air-borne treated groups (AL50 and AL90) were comparable to other studies with high-translucent zirconia [5,16]. It was expected that alumina abrasion with particles of larger size would result in a rougher surface, assisting micromechanical retention [32]. Indeed, the SEM micrographs and roughness measurements reported in our previous study showed that AL90 specimens had rougher surfaces (AL50 = 340.3 ± 49.2 nm) as compared to AL50 (AL90 = 1155.1 ± 97.76 nm) [27]. EDS analysis showed the increased Al and O content in both groups, probably due to the inclusion of Al_2_O_3_ particles onto the ceramic surfaces during alumina air-abrasion. However, statistically significant differences in SBS between AL50 and AL90 groups were not observed. In this way, the surface roughness profile from the different alumina particle sizes did not influence the bonding strength in the HTZ material, similarly to conventional zirconia, as previously reported [33].

The GB group presented very low shear strengths and very small percentages of the resin material remained on the surface, resulting in a high percentage of adhesive fracture mode. Based on our previous study and SEM analysis, it was observed that GB created a smooth surface with slightly higher toughness than the control (GB = 99.4 ± 16.6 nm, CL = 73.9 ± 10.7 nm) [27], likely because glass particles are much softer than alumina or zirconia. Therefore, another mechanism, irrespective of roughness, was responsible for these values. Possible contamination of the zirconia surface by the glass beads or incompatibility between them and the adhesive primer could explain the vertical drop in shear strength in this group, compared to CL that presented even lower surface roughness. Probably, very small glass beads—detected by EDS—could remain loose on the zirconia surface and interfere with the action of the primer’s phosphate monomers. As it was recently reported by Nagaoka group, zirconia surface contamination by residual silica particles may inhibit adequate bonding of the cement to zirconia [34]. On the contrary, Martins et al. [19] observed enhanced zirconia/resin cement bonding strength after air blasting with glass beads. The favorable results of this study might be explained by initial alumina air blasting and application of silane coupling agent for all specimens that was not performed in the present study.

Bonding is known to be affected by many factors, such as micro-mechanical retention, chemical adhesion, surface features, and the type of adhesives [35]. Recently, Yagawa et al. [36] tested the effect of different priming methods on bond strength between luting cements and a translucent zirconia material, without any micromechanical surface pre-treatment. It was found that the application of MDP priming agents created a durable bond strength between resin cements and translucent zirconia [36]. In the present study, we also applied an MDP-containing primer to achieve enhanced bonding. Our findings suggest that non-mechanically treated control specimens had a similar bonding ability to AL50 and AL90 groups and low percentage of adhesive failures. Low roughness and lack of surface contamination might have favored better surface wetting and thus could have improved the development of chemical bonds between zirconia primer and resin-based cement. In these cases, the shear bond strength seems to depend more on the inherent cement strength rather than on the surface roughness or that on unobstructed chemical bonding, irrespective of surface roughness, is necessary for effective bonding.

Apparently, surface treatments such as air abrasion often leave traces of contaminants on the material’s surface. Even though their amount is small, they can alter surface properties and compromise mechanical behavior as is the case with glass bead contamination. In this respect, ultrafast laser patterning is effective in creating an uncontaminated surface, as it can produce surfaces without any impurities. Besides, FS laser processing is a gentle and precise procedure, as it allows for the creation of different surface patterns without causing damage to the bulk material or significantly increasing the monoclinic phase [27], which makes it an appropriate method for HTZ ceramics surface conditioning. The feasibility to produce and alter zirconia surface patterning with the use of an FS laser is a wide-open field for future research, as many interesting retentive features can be created, with the aim of optimizing the bond strength.

Despite predictable and well-documented results, several limitations of this study should be mentioned. For example, the small number of experimental materials studied. Further research should include different HTZ materials with other commercial resin-based cements and conditions that mimic, with high precision, the oral environment such as cycling loading.

## 5. Conclusions

Under the limitations of this in vitro study, it can be concluded that airborne particle abrasion with alumina particles of different sizes (50 μm and 90 μm) and an FS laser, can yield similar bond strengths of HTZ ceramics to resin cements. However, none of the investigated treatments were superior to the others or compared to control polished zirconia surfaces. FS laser surface treatment of HTZ ceramics is a promising alternative method to enhance the mechanical retention of a resin cement as it is related to effective bond strengthening, without causing any surface contamination of zirconia specimens. On the contrary, abrasion with glass beads is not recommended for HTZ ceramics, as it presented significantly lower bond strengths compared to all other treatments.

## Figures and Tables

**Table 1:** Bonferroni Multiple Comparison tests of shear bond strength values among the groups.

Bonferroni Multiple Comparisons (Dependent Variable: Shear Bond Strength)						
(I) TREATMENT	(J) TREATMENT	Mean Difference (I–J)	Std. Error	Sig.	95% Confidence Interval	
					Lower Bound	Upper Bound
CL	FEMTO	−3.444	1.536	0.299	−7.979	1.091
	AL50	0.692	1.536	1.000	−3.842	5.227
	AL90	0.488	1.536	1.000	−4.047	5.023
	GB	8.601 *	1.536	<0.0001	4.066	13.136
FEMTO	AL50	4.136	1.536	0.099	−0.399	8.671
	AL90	3.932	1.536	0.139	−0.602	8.467
	GB	12.045 *	1.536	<0.0001	7.510	16.579
AL50	AL90	−0.204	1.536	1.000	−4.739	4.331
	GB	7.909 *	1.536	<0.0001	3.374	12.444
AL90	GB	8.113 *	1.536	<0.0001	3.578	12.648

* The mean difference is significant at the 0.05 level.

**Table 2:** Percentages of adhesive-type of failure (ADFM).

Group	ADFM (%)
CL	20% a*
AL50	30% a
AL90	30% a
GB	80% b
FEMTO	20% c

* Similar Latin characters indicate non-statistically significant differences between groups.
